# The grammar of emoji? Constraints on communicative pictorial sequencing

**DOI:** 10.1186/s41235-019-0177-0

**Published:** 2019-08-30

**Authors:** Neil Cohn, Jan Engelen, Joost Schilperoord

**Affiliations:** Department of Communication and Cognition, Tilburg University, P.O. Box 90153, 5000 LE Tilburg, The Netherlands

**Keywords:** Visual language, Emoji, Pictorial communication, Multimodality, Gesture, Grammar

## Abstract

Emoji have become a prominent part of interactive digital communication. Here, we ask the questions: does a grammatical system govern the way people use emoji; and how do emoji interact with the grammar of written text? We conducted two experiments that asked participants to have a digital conversation with each other using only emoji (Experiment 1) or to substitute at least one emoji for a word in the sentences (Experiment 2). First, we found that the emoji-only utterances of participants remained at simplistic levels of patterning, primarily appearing as one-unit utterances (as formulaic expressions or responsive emotions) or as linear sequencing (for example, repeating the same emoji or providing an unordered list of semantically related emoji). Emoji playing grammatical roles (i.e., ‘parts-of-speech’) were minimal, and showed little consistency in ‘word order’. Second, emoji were substituted more for nouns and adjectives than verbs, while also typically conveying nonredundant information to the sentences. These findings suggest that, while emoji may follow tendencies in their interactions with grammatical structure in multimodal text-emoji productions, they lack grammatical structure on their own.

## Significance statement

Emoji have rapidly become a prevalent part of everyday communication for millions of people worldwide. However, we still know little about the constraints guiding their comprehension, and how they function in communicative structures. Although emoji use a vocabulary, provided through the digital interface of smartphones and computers, it is less clear whether a grammatical system guides how emoji are sequenced. In addition, we still only have a growing understanding of how emoji interact structurally with the properties of written text. This study therefore asked participants to communicate with each other using only emoji (Experiment 1) or to explicitly replace emoji for words in their sentences (Experiment 2). Although we found that participants consistently use emoji for expressive and communicative means, their sequencing properties are limited in their complexity. In addition, while they typically add nonredundant information to the meaning of a sentence, when emoji are substituted into sentences they are used more often to replace nouns and adjectives than verbs. Overall, these findings suggest that emoji make an effective communicative tool, but the system guiding their sequencing is limited in complexity.

## General introduction

Emoji use has exploded in recent years in interactive digital communication. These simple images, now integrated with most digital keyboards, have become a mainstay of messaging and social media. Their broader cultural significance has rocketed them into the popular and academic spotlight, with proclamations that emoji are an ‘emerging language’ (Danesi, 2016; Lebduska, 2014; Lu et al., 2016) and increasing advocacy for them in research (Kaye, Malone, & Wall, 2017). Clearly, emoji provide a visual vocabulary (albeit limited) that enable users to communicate. However, only a few studies have examined the properties of their sequential patterning—i.e., their ‘grammar’ (McCulloch & Gawne, 2018). Thus, we ask: *do emoji use a grammar and how might they interact with the grammar of sentences?* Here we report two experiments asking participants to converse using either only emoji (Experiment 1) or in sentences with emoji substituted for words (Experiment 2).

The current vocabulary set of emoji grew out of emoticons, the facial images created by mixing letters and punctuation to form different faces such as :) and ;). In the late 1990s, a set of pictorial signs were then created in Japan deemed ‘emoji’—picture (*e*) character (*moji*)—which became integrated into digital keyboards. Many emoji were pulled from the conventional depictions of emotions used in Japanese manga (Moschini, 2016), as evident in the nose bubble for sleep , shade on the forehead for dread , and the large sweat drop on the forehead for anxiety  (Cohn, 2013a; Cohn & Ehly, 2016). As people began using emoji, emoticons reduced in prevalence (Pavalanathan & Eisenstein, 2016) or became automatically converted into emoji, and now emoji have become a common part of interactive digital communication. This emoji vocabulary is now integrated into operating systems as a relatively fixed list controlled by the Unicode Consortium, a conglomerate of technology companies that decide how computers encode typed information. Thus, variants to emoji and creation of new emoji are largely decided by this governing body.

Many of the studies so far on emoji have focused on the frequency of emoji use (Lu et al., 2016), their communicative function (Kelly & Watts, 2015) and comprehensibility in conversations (Berengueres & Castro, 2017), how they differ in interpretation based on variance in emoji keyboards (Miller et al., 2016), and their general semantics in relation to each other (Ai et al., 2017; Barbieri, Ronzano, & Saggion, 2016). Additional work has stressed the functions that emoji serve for conveying unseen facial information, to control a conversation, or to convey a sense of playfulness or humor (Kelly & Watts, 2015). Psycholinguistic research has also shown that emoji at the end of a sentence can effectively convey irony, eliciting similar brain responses as markers of verbal irony (Weissman & Tanner, 2018).

Other studies have classified the ways that emoji appear relative to sentence contexts. For example, using a WhatsApp conversation corpus in Swiss-German, emoji have been observed at the end of sentences to clarify their tone or mark their ending far more than they substitute for lexical items or homophonous letters (Duerscheid & Siever, 2017). Other work using a Twitter corpus has substantiated the prevalence of emoji used to enrich or clarify the tone of the preceding sentence (Na'aman, Provenza, & Montoya, 2017). These were found to be far more frequent than emoji substitution for function words and punctuation, or content words like nouns and verbs. This is consistent with early work on emoticons claiming that they convey a “stance” placed at the ends of sequences (Schnoebelen, 2012).

In these distributions, emoji primarily combine with language in multimodal interactions in ways similar to co-speech gestures (McCulloch & Gawne, 2018), where manual expressions combine with spoken language (Goldin-Meadow, 2003a; McNeill, 1992). Concurrent gestures occur in temporal correspondence with speech (Hutchins & Nomura, 2011)—they are linked by virtue of occurring at the same time (Clark, 1996). Emoji are not produced in a spatially separate channel from written language (i.e., both are typed linearly), and thus cannot benefit from simultaneity. However, they often fall before or after an utterance (Cramer, de Juan, & Tetreault, 2016; Kelly & Watts, 2015; Markman & Oshima, 2017; Zhou, Hentschel, & Kumar, 2017), serving as an emotionally charged punctuation (Duerscheid & Siever, 2017; Na'aman et al., 2017), possibly to play a role analogous to concurrent gestures. Component gestures or language-like gestures are those that substitute for a word in sentence (Clark, 1996; McNeill, 1992), as in “She caught a *<wide gesture>* fish” where the gesture substitutes for the word “huge”. Such substitutions of one modality into the grammar of another occurs in relationships between most expressive modalities, be it speech and gesture or text and image (Cohn, 2016).

When emoji are used in sequences, they primarily use repetition, or reduplication, of the same emoji (McCulloch & Gawne, 2018; Tatman, 2018). When they are not repeating, emoji prevalently cluster with others that maintain a common semantic field, or theme (McCulloch & Gawne, 2018). Some scholars have focused on the use of emoji as rebuses or calquing—where emoji substitute directly for words that sound like the names of the images depicted, although without the meaning (Danesi, 2016). For example, Danesi (2016) gives an example of how the utterance “bombshell bikini” could be formed with   , with the first two emoji acting as rebuses, but it can only be interpreted appropriately by English speakers familiar with the expression.

Other speculation about emoji sequencing has revolved around the order of possible grammatical roles (e.g., ordered parts-of-speech such as subject-object-verb (SOV) versus SVO orders), and whether emoji-only utterances use grammar-like categorical roles and can differentiate between agents, patients, objects, and actions/verbs. Indeed, observers can rapidly and efficiently extract such roles from complex visual scenes (Hafri, Papafragou, & Trueswell, 2012; Hafri, Trueswell, & Strickland, 2018), and from the drawn images within narrative sequences such as comics (Cohn & Paczynski, 2013; Cohn, Paczynski, & Kutas, 2017). Thus, it is conceivable that event roles are also recognized in emoji sequences. Nevertheless, purely emoji sequences may rely on conceptual categories (such as agent, patient, act) and not purely grammatical ones (subject, object, verb), the latter of which is defined by distributional tendencies within a system rather than their relational semantic traits.

The question then is, do emoji use categorical roles—be they semantic or grammatical—and, if so, what order(s) might be prevalent? In previous work on nonverbal gesturing, Gershoff-Stowe and Goldin-Meadow (2002) asked whether language-like structures would emerge if speaking participants (who did not use a sign language) communicated using only gestures. They found that, over the course of a conversation, participants converged on more systematic gestured vocabulary, and demonstrated certain regularities of patterning. Specifically, participants appeared to prefer an agent-patient-act order of semantic roles (i.e., doer of action, receiver of action, action—such as girl-pizza-eat), despite being speakers of English, a language with a canonical subject-verb-object structure. This agent-patient-act order was consistent with those prevalently produced by deaf children born to hearing parents, who invented their own manual communication system regardless of the grammatical order found in the (unheard) language of their environment (Goldin-Meadow, 2003b; Goldin-Meadow & Mylander, 1990).

Following this work, one possibility is that emoji follow the consistent agent-patient-act order observed in studies of nonverbal gesturing. Indeed, in another experiment in their study, Gershoff-Stowe and Goldin-Meadow (2002) found that participants used the agent-patient-act pattern when asked to order images to construct a spatial scene used to describe an animated action. In addition, even children as young as 3 years old prefer ordering causal relations of images with entities prior to actions (Gelman, Bullock, & Meck, 1980). This agent-patient-act advantage may arise because agents are a ‘core’ aspect of cognition (Strickland, 2016), which serve to more efficiently build event structures than patients (Cohn & Paczynski, 2013).

Nevertheless, another possibility could be that emoji orders would follow the grammatical order of a person’s spoken language (e.g., SOV versus SVO). Some work has found that ordering of images also may exhibit features of a participants’ spoken language (Vastenius, van de Weijer, & Zlatev, 2016), and gesture utterances are more likely to follow an SVO order if they follow a known vocabulary, rather than the SOV order of improvised communication (Marno et al., 2015). Indeed, it has been argued that linguistic systems prefer an SVO order, despite a pervading cognitive bias for SOV orders in improvised communication (Langus & Nespor, 2010), possibly motivated from basic semantic heuristics like agent-first or entities-before-actions (Cohn & Paczynski, 2013; Jackendoff & Wittenberg, 2014). Following the word order of a native language may also be facilitated from the predictive emoji generation of keyboards, allowing people to type words and convert them to suggested emoji. Thus, might emoji involve grammatical patterns similar to basic sequencing observed across domains, or similar to a speaker’s spoken language?

Our research question thus asked whether participants use a grammatical structure to produce emoji, and how emoji might interact with verbal syntax in substitutions for words. We reasoned that such grammatical structure—if there was any—would most likely emerge if used as the primary mode of communication. We therefore modified Gershoff-Stowe and Goldin-Meadow’s (2002) methods by asking participants to have conversations using emoji as their dominant communicative modality. Participants communicated digitally in conversations where one speaker could use text and the other replied only in emoji or where both could only use emoji (Experiment 1), or where both could use text, but had to substitute emoji into each sentence (Experiment 2). Our analysis then examined the ways in which emoji exhibited sequencing patterns and/or interacted with the written language syntax. Although the experiments were run as part of a single session, each experiment had independent hypotheses, and thus we report them separately.

## Experiment 1: emoji sequencing

Our first question asked whether emoji exhibit complex grammatical properties such as patterned parts-of-speech or phrase structure when used productively in isolation. While some studies have examined the interaction of emoji with sentences (Duerscheid & Siever, 2017; Na'aman et al., 2017), few studies have examined the sequential properties of emoji utterances in their own right.

The question whether emoji sequencing would follow language in part assumes that emoji sequencing will be like—or will develop beginning like—those of matured verbal languages. However, not all sequencing uses complex grammatical structure, even in full languages. Thus, analysis of ‘grammar’ in emoji can benefit from recent nuanced taxonomies of grammar in language. Jackendoff and Wittenberg (2014) propose a new ‘hierarchy of grammars’ based on how a system maps form and meaning with basic ordering principles. This hierarchy is proposed as a more ecologically valid taxonomy of grammar than the classic Chomskyan hierarchy (Chomsky, 1956). Rather than characterize idealized logical forms like the Chomskyan hierarchy, Jackendoff and Wittenberg’s approach characterizes properties of actual communicative systems in the world, ranging from those not reaching the full designation of a ‘language’ (proto-languages, stages of children’s language development, homesigns, etc.) to fully robust linguistic systems.

At the lower levels of Jackendoff and Wittenberg’s (2014, 2017) hierarchy, the “grammars” themselves have little influence, and utterances are motivated by the semantics of the units themselves. The hierarchy begins with one-word grammars, in which each expression stands alone with no sequential properties. In speech, various words with nonsyntactic properties use one-word grammars, such as ‘gadzooks’, ‘ouch’, or various ideophones like ‘pow!’ or ‘honk!’ (Dingemanse, 2012). These utterances play no grammatical roles (like nouns or verbs), and cannot be put into the syntax of a sentence without being marked. The manual motions of co-speech gesture also usually use a one-word grammar, since gestures appear roughly once per spoken clause (McNeill, 1992). A two-word grammar exhibits similar basic properties as one-unit grammars, but involves two utterance units, as is found in the two-word stage of language development, and in pivot grammars.

Linear grammars^1^ involve a series of juxtaposed units, with no internal phrasal structure. These structures allow for units to be concatenated in strings of indefinite length. Jackendoff and Wittenberg argue that linear grammars are in fact fairly prevalent, including in Pidgins, emerging sign languages, and some widely used spoken languages. A simple phrase grammar then allows for the embedding of a sequence within an utterance, giving way to basic phrase structures.

Orthogonal to this, units in an utterance can play categorical roles defined by distributional tendencies in a system (e.g., ‘parts of speech’ like nouns and verbs), in what Jackendoff and Wittenberg call a ‘part-of-speech simple phrase grammar’. It is important to note that these grammatical roles are not defined by semantics. Semantic roles such as agents and patients (Dowty, 1991; Gruber, 1965)—i.e., the doer and receiver of an action—occur even in the absence of a more complex grammatical structure, and may operate on the basis of simple heuristics, like ‘Agent-First’ or ‘Entities-Before-Actions’ (Cohn & Paczynski, 2013; Jackendoff & Wittenberg, 2014), which may give way to the widespread agent-patient-act order. Finally, the highest level of grammatical complexity is a recursive grammar, which allows for phrases to embed within other phrases.

The Jackendoff and Wittenberg (2014) hierarchy provides a more nuanced and ecological characterization of grammatical patterning than other approaches, and similar taxonomies have been proposed for the neural encoding of patterns across domains (Dehaene, Meyniel, Wacongne, Wang, & Pallier, 2015). We propose that this overall model can characterize the sequencing patterns found in emoji. Previous work has found that complex visual narrative sequences of images, as found in comics, use recursive grammars for narrative with hierarchic structures involving categorical roles (Cohn, 2013b), and behavioral and neurocognitive measures have shown evidence that such a structure is processed by similar mechanisms as that of syntax (Cohn, Jackendoff, Holcomb, & Kuperberg, 2014; Cohn, Paczynski, Jackendoff, Holcomb, & Kuperberg, 2012). Thus, we have precedents for believing that grammar in sequential images is possible; the question is whether emoji demonstrate such features.

Within the context of the Jackendoff and Wittenberg hierarchy, one-unit grammars might be a single image utterance, such as a sentence-ending emoji expressing an emotive stance. A linear grammar would be a sequence of images with only juxtaposed relationships between images, such as ‘visual lists’ which share semantic associative features (a sequence of animal images) or an unrelated list bound by a superordinate category. A linear grammar would also appear in a stepwise temporal sequence of events (x then y then z…) that has no segmentation or categorical roles of units. Reduplication (i.e., repetition) of an emoji would also use a linear sequence, as a flat list-like structure. For examples, see Table 1.

**Table 1:** Different analyzed types of emoji sequencing

Emoji type	Definition	Example
One-unit grammars		
Formulaic expressions	Emoji serving conversational functions (yes, no, hmm)	= hello
Responsive emotions	Emoji used for feelings or emotions	, , ,
Affixation	Attachment of two emoji to create a larger single unit	= running person
Whole image	Combination of emoji to create a single “picture”	= person running to a ferris wheel
Linear grammars		
Reduplication	Repetition of the same emoji	
Semantic list	Emoji related by a semantic associative field	= “dress nice”
Unrelated list	List of emoji with no intrinsic semantic relationships	= “I see myself graduated, animals, house, job”
Temporal sequence	A linear sequence of events	= “In the morning see a cow, then chicken, then…”
Categorical grammars		
Three-unit (SOV, SVO, etc.)	Three-unit sequence of emoji playing “grammatical” roles	= “(I) hope soon to get a dog”
Two-unit (SV, SO, OS, OV, VS, VO)	Two-unit sequence of emoji playing “grammatical” roles	= “(I) don’t want to get married”
Simple phrase grammars		
Embedded sequencing	Sequencing where one grouping was embedded in another grouping	= A couple in love [] goes to the ferris wheel []
Other classifications		
Metonymy	An emoji with a related meaning to the actual message	, = work/job
Rebus	Use of an emoji for its phonological correspondence unconnected to its visual meaning	= book a flight

Examples come from produced emoji-only utterances in Experiment 1, and participant annotations are in quotes

*O* object, *S* subject, *V* verb

All of these types of sequencing would be expected to appear without the need for categorical roles or constituent structure. True categorical roles such as nouns and verbs (and thus also subjects and objects) do not appear until higher levels of grammars, as would phrase structures. It is also possible that emoji usage will not prefer grammatical roles determined by verbal syntax, such as nouns and verbs; complex sequential images have been previously shown to use *narrative* roles defined by distributional tendencies in a sequence (Cohn, 2013b, 2014). Thus, within the Jackendoff and Wittenberg hierarchy, if emoji display consistent patterns of categorical roles, it would reflect higher levels of grammatical structuring. Nevertheless, given prior observations of the limited nature of emoji utterances (McCulloch & Gawne, 2018), we predicted broadly that only minimal structure would emerge in the ‘grammatical’ properties of emoji sequencing. This should therefore stay at the lower levels of the Jackendoff and Wittenberg (2014) hierarchy: linear grammars without grammatical roles or codified word orders.

### Methods

#### Participants

Sixteen participants (2 male, 14 female, mean age 25.4 years, range 21–31 years) from Tilburg University participated in the experiment in pairs. Participants were informed of the minimal risks involved in the study, were given the ability to withdraw at any time, and all gave informed written consent prior to the experiment. The sample was culturally diverse and, although all participants spoke English, they also spoke several different languages including Albanian, Dutch (five participants), French, German, Greek, Indonesian, Italian, Japanese, Korean, Mandarin (two participants), Norwegian, Portuguese, and Spanish.

Based on post-test questionnaires, 31.25% of the participants reported to have previous experience with the chat application used in the experiment (Google Hangouts). On a five-point scale (1 = ‘I use emoji rarely’; 5 = ‘I use emoji very often’), participants used emoji at an average frequency of 3.75 (SD = 0.93). Our post-test questionnaires also asked about participants’ context and use of emoji in daily digital communication, with a box allowing for open-ended responses. Their responses suggested that they use emoji primarily in the context of direct messaging with friends and family, often with the purpose to make conversations “more casual”, to “express feelings”, or “to avoid misinterpretation”.

#### Procedure

Participants took the study in pairs. Each participant was placed in a soundproof chamber where they could not interact face-to-face with their experimental partner. Participants were then asked to communicate in brief digital conversations using Samsung tablets with the Google Hangouts software application to facilitate communication in words and in emoji. This application was used because it facilitated the export of chats for later analysis and could be used without a phone number. We used laboratory-based accounts and, after the experimental data was saved locally, we deleted all conversations. Conversations were broken up into four different rounds, each with a different topic and restriction of emoji use. Rounds lasted roughly 7–9 min. Some participants were given extra time prior to the experimental rounds to acquaint themselves with the software and tablets.

The tasks of the first three rounds of the experimental session pertained to Experiment 1, while the fourth round pertained to Experiment 2. In the first round of conversation, one participant was allowed to use written words, while their partner could only use emoji and punctuation (which could potentially facilitate constituent structure and/or emotion with ‘!’ or ‘?’). In the second round, the participants switched roles, with the emoji-only person in round 1 using words and vice versa. In both rounds, the partner using written words was not restricted from using emoji to also supplement their conversations, though it was not specified. In the third round, both participants were asked to only communicate in emoji, emoticons (e.g., arrows -->), and punctuation without any written words or letters. However, emoji that incorporated words in them were acceptable (e.g., ). The fourth round asked participants to communicate with sentences that substituted emoji for words. As this round is reported in Experiment 2, we provide more details about this task below. The order of tasks across experimental rounds was kept the same across participants.

Unlike Gershoff-Stowe and Goldin-Meadow’s (2002) study, we did not have the participants attempt to describe eliciting stimuli to each other, but rather we sought to create a more naturalistic communicative exchange with open-ended conversation. In order to facilitate these conversations between our participants, we provided conversational prompts with topics of discussion that were optimized for the limited emoji vocabulary set. According to emojipedia.org, the Google emoji lexicon in 2016 used in the study comprised 1796 emoji (Google Android 7.0) with categories of smileys and people, animals and nature, food and drink, activity, travel and places, objects, symbols, and flags. Based on these categories, our conversational topics included ‘the perfect date’, ‘travelling’, ‘future plans’, and ‘zoo visit’. To encourage our participants to communicate, we used leading questions for each topic such as “Ask the other person to help you plan the perfect date”, “Get the other person to tell you about their future plans over the next ten years”, and so forth. Additional guiding questions were also offered, such as “What would be a perfect date for you?” and “How would you describe your perfect partner?”. The order of topics of discussion was rotated between rounds across pairs of participants using a Latin square design. This ensured that, across all participants, each topic was used an equal number of times in each experimental round.

After each session, participants filled out a post-test questionnaire inquiring about their attitude towards different modes of communication, their use of emoji, their enjoyment of the chat sessions, the comprehensibility of conversations, and their fluency in different languages. Each participant also annotated a printout of their conversations, describing 1) what they meant with their messages and 2) how they perceived the messages sent by the other participant. Altogether, experimental sessions lasted 30–60 min.

#### Annotation

We analyzed emoji across the major categories of the Jackendoff and Wittenberg (2014) hierarchy, and subcategorized sequencing types based on the prior literature. Our unit of analysis in the annotation was a ‘segment’, defined as any span of emoji that formed a recognizable grouping. This was identified first as an isolated line of conversation set apart by a message break, or, less frequently, as multiple segments within a single line of conversation. These segments were determined first by consulting participants’ own annotations and second by our analytical categories. Annotations were not mutually exclusive, and segments could fall into multiple types, if appropriate. Below we describe these annotations, and provide examples in Table 1.

The most basic use of emoji would be constrained to one-unit grammars. Emoji that are similar to formulaic expressions conveyed emotions such as “yes”, “no”, “huh”, “hmm”, and “sure”, comparable to gestural emblems such as waving hello (McNeill, 1992). Responsive emotions are emoji used for expressing feelings and emotions (, , ) either in isolation or combined with another sequence of emoji or text. Our analysis of one-unit grammars was specifically constrained to these expressions and/or other single unit expressions.

‘Affixation’ refers to combining emoji to create a combinatorial meaning that retained the appearance of a ‘single image’. For example, a puff of smoke emoji  is similar to the ‘visual affix’ of a motion line, as used in comics (Cohn, 2013a), which could attach to a running person  to enhance the sense of motion and speed: . As in morphology, visual affixation stays at the level of a single unit, although creating a more complex unit. Like affixation, ‘whole image’ emoji were those that appeared to create a single iconic image-unit, but without affixation (e.g., , to iconically show that the man chased the car). These cases rely on the natural combinatorial properties of visual images (Cohn, 2013a), yet still might allocate ‘categorical’ roles of the relational semantics. Thus, these types of representations would be annotated as a ‘whole image’ because of their iconicity, but also possibly as reflecting a categorical grammar (see below) if that image also involved relational semantic roles.

As in Jackendoff and Wittenberg’s hierarchy, we considered linear grammars as juxtaposed relations of units bound only by semantic relations. Unordered linear grammars were sequences where the particular order of units did not matter for the meaning being conveyed. Reduplication simply repeated the same emoji multiple times, particularly as a listing for emphasis (e.g., “I *really really really* like it”). Semantic lists were comprised of emoji that maintained a common semantic associative field (e.g., emoji representing office furniture, list of different animals, and so forth). An unrelated list was a sequence of emoji that had no unifying semantic field, but may have cohered through their context in the conversation. An ordered linear grammar was a linear sequence where the order of images mattered for the meaning. This was primarily a temporal sequence*,* which conveyed sequential events which occurred one after the other.

We considered sequences where units had a clear word-like order where emoji played relative roles as belonging to categorical grammars (Jackendoff & Wittenberg: ‘part-of-speech simple phrase grammar’). These sequences could be either three units long (SVO, SOV, etc.) or two-units long (SV, SO, OS, OV, VS, VO). Although we recognize the difference between semantic categories (agents, patients, actions, sources, goals) and grammatical ones (subjects, objects, nouns, verbs), for simplicity we collapsed the semantic notions of agents, sources, etc. to “Subjects”, patients, goals, etc. to “Objects”, and actions to “Verbs.” This was also done to give emoji the benefit of the doubt in generalizing across semantic roles, rather than restricting them solely to doers and receivers of actions (agents and patients). Along these lines, we took a generous approach to our assignment of categorical roles, using this coding for any emoji with a possible relative role to another emoji that appeared functional and did not otherwise fall into our other categories.

We defined emoji using a simple phrase grammar as any that embedded one grouping of emoji within a larger sequence. Embedded constituents could have the characteristic of other patterning (e.g., combinations of a semantic list within a larger temporal sequence, reduplication within semantic lists, and so on). We also counted the level of embedding that was used (i.e., a single constituent within a larger sequence would be one level of embedding, while a constituent within a constituent within a sequence would be two levels). We found no need for a recursive grammar, as it did not appear in any utterances.

Finally, some traits fell outside the classification of grammatical levels. A metonymic emoji conveyed a meaning through a causal or conceptual relationship to the intended message. Here, metonymic emoji characters (usually material objects) were used to represent more abstract concepts or words, for example  or  being metonymic for “work” or “job”, or  referring to the act of graduating. A *Rebus* was similar to Danesi’s (2016) notion of calquing, where the sound quality of an emoji is used to represent a word; for example,  to mean ‘book a flight’.

#### Data analysis

Our analysis examined all emoji-only utterances from experimental session rounds 1–3. For each participant, we calculated a mean for how many times each category under analysis was used out of all of their utterances. Note that a single utterance could be coded for multiple traits. We first asked whether certain patterns were used at a significant rate overall. Using a one-sample *t*-tests, we compared the means for each category against the frequency rate of .043—the chance of a category occurring 1 time out of the 23 total categories analyzed. We here focus on those that appeared significantly more frequently than this threshold. We then asked whether there were differences between the frequency rates of targeted categories. Paired *t*-tests were used for comparisons between two categories alone, while repeated-measures analysis of variance (ANOVA) were used to examine more than two categories, followed by post-hoc comparisons with a Bonferroni correction. Finally, planned correlations were used to assess the relationship between different categories (e.g., SVO or VO orders) or with participants’ background expertise (e.g., speaking an SVO language with use of SVO orders).

### Results

**Fig. 1:**
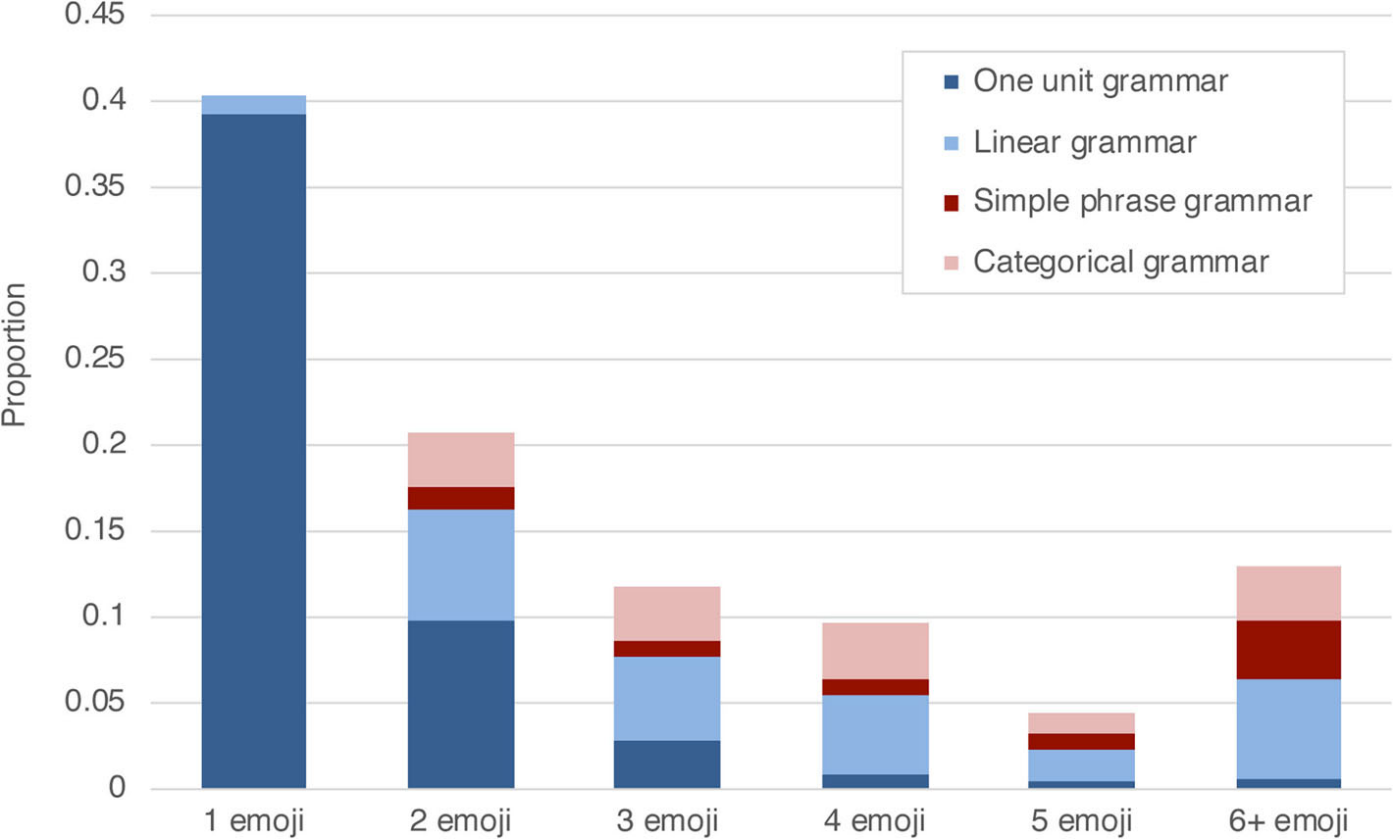
Number of emoji used in emoji-only utterances and the types of sequencing complexity used in those utterances. Note that one-unit grammars could comprise more than one emoji (see Table 1)

We analyzed 286 utterances, within which participants averaged 17.8 (SD = 6.1) utterances per conversation. Participants used an average of 1.28 (SD = 0.23) segments per utterance, and 2.79 (M = 1.13) emoji per utterance. A significant effect was found for comparing the length of participants’ emoji utterances; F(5,75) = 16.7, *p* < .001. Here our results followed Zipf’s law that the length of units is inversely proportional to their frequency (Zipf, 1935): utterances with one emoji were used the most, followed by two emoji through to five emoji, with slightly more for six or more emoji in an utterance (a grouped category), as depicted in Fig. 1. Polynomial contrasts revealed that this was a linear trend; F(1,15) = 22.05, *p* < .001.

As depicted in Fig. 1, complexity of grammar varied across utterance length. This demonstrates that where there necessarily is a relationship going from a length of 1 to longer utterances—indeed, some grammar types require at least two or more units—no systematic relationship persists between the length of utterances and the types of sequencing complexity among longer utterances (i.e., three units and longer).

We next compared across the different levels of the hierarchy of grammars, where we found that overall levels differed in their frequency; F(3,45) = 55.0, *p* < .001. The rates of each level descended according to the levels of the hierarchy: one-unit grammars (M = 0.82, SD = 0.28) were used more than all other types, followed by linear grammars (M = 0.45, SD = 0.27), categorical grammars (M = 0.18, SD = 0.15), and simple phrase grammars (M = 0.09, SD = 0.1). All types differed from each other (*p* < .005) except for categorical grammars and simple phrase grammars (*p* = .193).

As a follow up analysis, we asked could it be that, although simpler grammars pervaded overall (i.e., not categorical or nonphrase structure grammars), the complexity of sequencing increased as participants proceeded through the experiment? Might they have become more systematized and complex as they habituated to using only emoji to communicate? Since both variables ‘level of grammar’ and ‘serial order of utterances within each session’ are ordinal scores, we performed a Spearman correlation to find out whether complexity increased as participants proceeded through the experiment. However, we found no significant effect of serial order on the complexity of the grammar (*p* = .274).

**Fig. 2:**
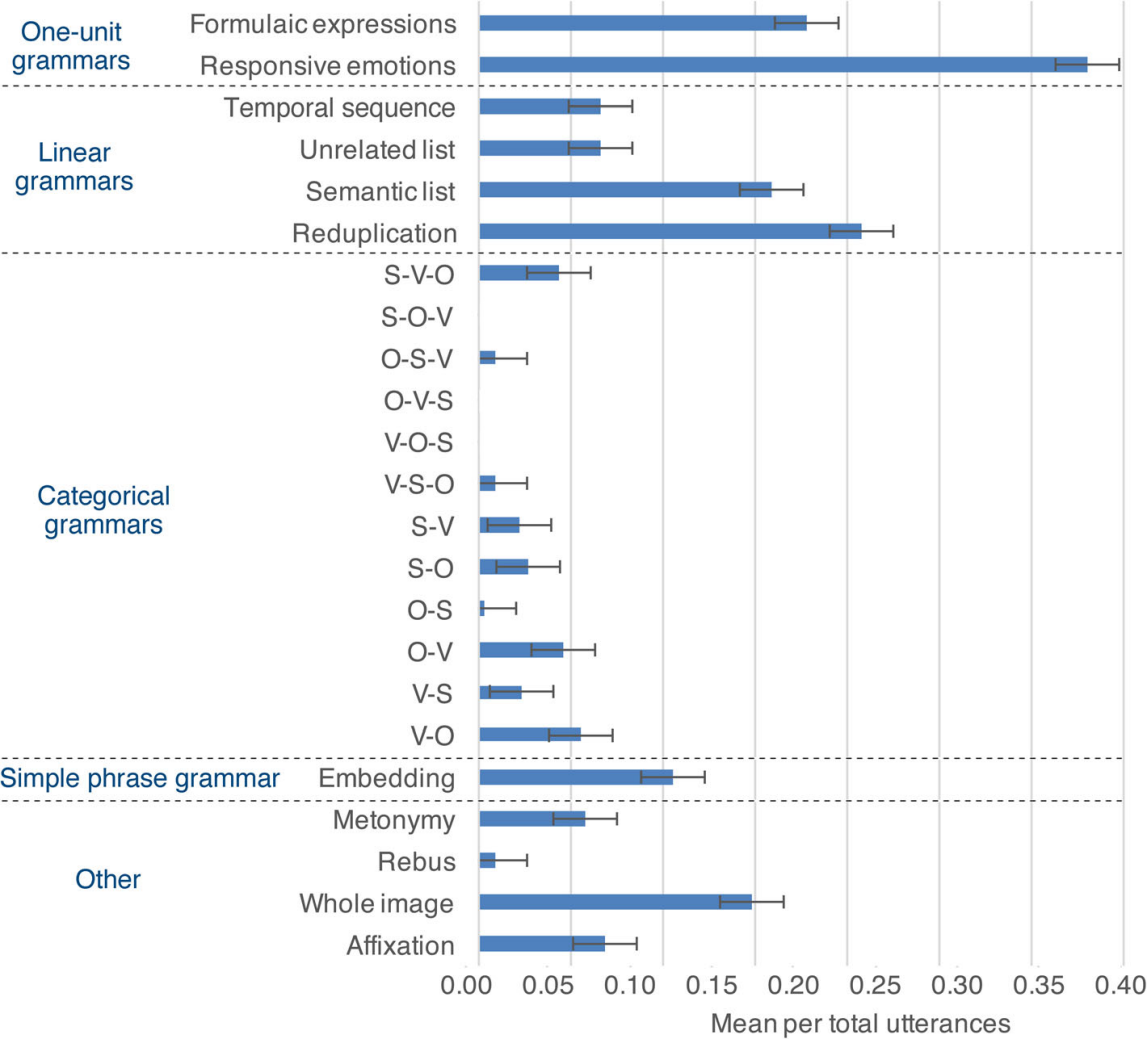
Types of utterances used in emoji-only conversations. Error bars depict standard error. O object, S subject, V verb

Additional analysis found variation between types of emoji within these broad grammar types, which are summarized in Fig. 2. Within one-unit grammars and ‘other’ classifications, formulaic expressions, responsive emotions, and whole images were used at a frequency greater than chance (i.e., higher than .043) (all *t* > 3.3, all *p* < .01). Metonymic and affixation emoji were used near the threshold but did not exceed it. We found only a single example of rebus emoji uttered in the whole experiment (the “book a flight” example). These types of emoji patterns differed overall; F(5,75) = 27.6, *p* < .001. This difference arose because responsive emotions were used more than all other types (*p* < .001), and indeed were the most frequent type of emoji utterance in our experiment. Formulaic expressions, the third most frequent type in the experiment, were also greater than metonymies and rebuses (*p* < .05), but did not differ from whole images or affixation.

We further investigated where responsive emoji were placed when they accompanied longer sequences. These emoji differed in whether they appeared before, after, or within a sequence; F(2,30) = 7.2, *p* < .005. Emoji surrounding a sequence—both before (M = 0.09, SD = 0.09) and after (M = 0.05, SD = 0.05) other emoji—occurred more often than those placed within (M = 0.01, SD = 0.03) an emoji sequence (all *p* < .001).

Of the linear grammars, semantic lists and reduplication both exceeded the threshold of chance for appearing (all *t* > 0.35, all *p* < .005). Temporal sequences and unrelated lists both appeared near the threshold, but did not exceed it (all *p* > .136). These types also differed significantly from each other; F(3,45) = 7.0, *p* < .005. This arose because reduplications and semantic lists were used more than temporal sequences and unrelated lists (all *p* < .05), which did not differ. With reduplications, participants averaged 2.9 (SD = 1.78) repeated emoji per utterance.

Categorical grammars both of two-units (e.g., subject-verb) or three units (e.g. subject-object-verb) were all used fairly infrequently. Overall, two-unit sequences (M = 0.18, SD = 0.16) were used significantly more often than three-unit sequences (M = 0.06, SD = 0.09), *t*(15) = 2.5, *p* < .05. In addition, two-unit sequences were used more often than our threshold of significance, *t*(15) = 3.4, *p* < .005, but three-unit sequences were not (*p* = .446). However, none of twelve possible two- or three-unit patterns exceeded the threshold of significance in frequency overall (all *p* > .173). In addition, no utterances were recorded for the patterns of SOV, OVS, or VOS, while OSV and VSO orders were both under a rate of .01 (all *t* < −4.9, all *p* < .01). SVO orders (M = 0.043, SD = 0.07) appeared at the same rate as the threshold of chance (.043) (*p* = .984).

When including all six three-unit category sequence types, a difference appeared between patterns; F(5,75) = 4.37, *p* < .002. However, we found no significant differences in our pairwise comparisons (all *p* > .381). Given that three of these sequence types yielded no utterances at all (SOV, OVS, or VOS), we also analyzed only the three types that had utterances. Here, we observed only a near significant difference between SVO, OSV, and VSO orders; F(2, 30) = 3.3, *p* = .051.

Differences did appear between two-unit category sequences; F(5,75) = 2.5, *p* < .05. Post-hoc analyses revealed no significant differences between orders except between OS orders (the most infrequent type) and VO orders (the most frequent type) (*p* < .05). No correlation appeared between participants’ rated fluency in SVO languages and the rate of that order (*p* = .650), though a trending correlation appeared with the rate of VO orders, *r*(14) = −.481, *p* = .059, but not SV orders (*p* = .514). A positive correlation did appear between SO orders and SOV fluency, *r*(14) = .773, *p* < .001, but not OV orders (*p* = .509).

**Fig. 3:**
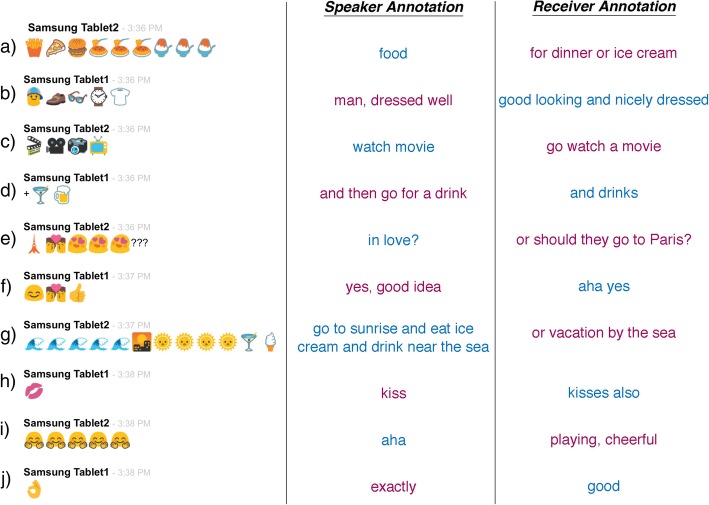
An excerpt from an emoji-only conversation between two female participants with the prompt of ‘What is a perfect date?’, along with post-experiment annotations given by the speaker and receiver of the messages. Note the prevalence of reduplication of emoji and semantically related lists of emoji

Emoji utterances using a simple phrase grammar were those that used embedding in the sequence. These utterances occurred more often than our threshold; *t*(15) = 2.6, *p* = .05. When such phrases occurred, they used a single level of embedding, typically a composite whole image placed within a larger sequence, or reduplication within a semantic list (see Fig. 3a). Some participants would perform such embedding more than once in an utterance, but a maximum of three times.

Finally, we assessed the ‘success’ of participants at communicating using only emoji. In post-test questionnaires, participants indicated an average difficulty of understanding emoji-only utterances, rating them an average of 3 (SD = 1.1) on a five-point scale (1 = very easy, 5 = very difficult), which was slightly higher than their assessed ease of creating emoji-only messages (M = 2.38, SD = 0.8). Nevertheless, a comparison of participant annotations suggested that only 17.5% utterances overall were misunderstood, with a rate of 3.12 misunderstood utterances per person. Here, ‘understanding’ was taken as the assessed comprehension of a gist of an utterance, even if some of the individual emoji may have been misinterpreted.

### Discussion

This experiment asked participants to communicate using only emoji, and then examined their sequencing for ‘grammatical’ patterning. Overall, participants used fairly simple constructions, exhibiting features of one-unit and linear grammars. The most frequent emoji utterances were those expressing responsive emotions (such as the final “OK” gesture, see Fig. 3j). This maintains the idea that emoji function as a nonverbal expression for conveying moods and attitudes, particularly at the end of sequences (Fig. 3f), which is reinforced by the prominence of facial expressions in the emoji vocabulary set. Formulaic expressions were also frequent, reflecting the use of emoji to uphold social and discourse markers (‘yes’, ‘no’, ‘hmm’).

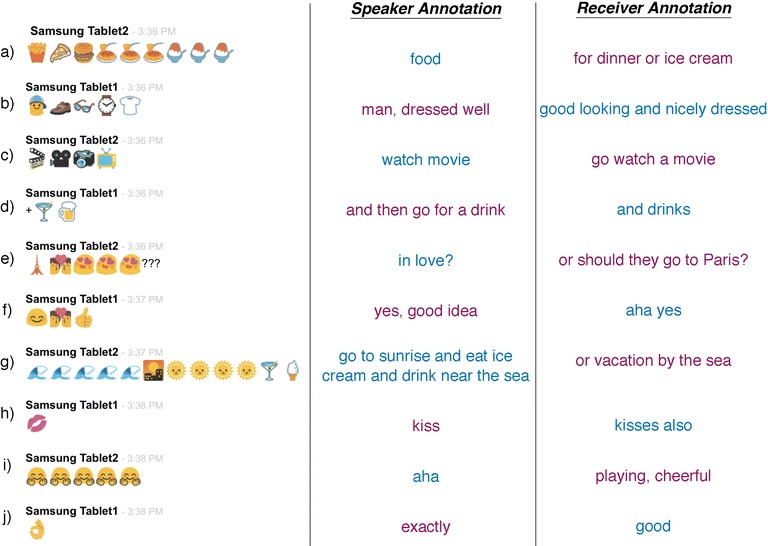



Linear grammars also appeared fairly frequently, particularly reduplication and lists. Reduplication was used as a signal for emphasis or increased magnitude, which is a common function for repetition of morphemes across languages (Moravcsik, 1978). For example, the repeated smiling faces in Fig. 3i convey an increased emphasis of the happy response (*really* nice), while the repeated food items in Fig. 3a convey the magnitude of the amount of food being eaten (*a lot of* pasta and dessert). The prevalence of these reduplications is consistent with corpus analyses suggesting that repeated emoji are the most frequent type of sequencing (McCulloch & Gawne, 2018; Tatman, 2018). Semantic lists also appeared frequently, with participants stringing together several emoji of a related semantic field, as in Fig. 3b using various clothing items to describe a well-dressed man. This is consistent with findings by McCulloch and Gawne (2018) who found in a corpus of over a million emoji that all of the top 200 sequences of nonrepeating emoji were thematically linked. The example conversation in Fig. 3 predominantly uses reduplication and semantic lists, and indeed combines these strategies (Fig. 3a).

One of our more surprising findings was the minimal frequency of emoji utterances that used categorical roles. Despite debates about word orders in emoji sequences (Tatman, 2016), relative to the languages of their speakers, we found that such utterances—regardless of pattern—constituted a minimal proportion of the utterances. Within such utterances, participants preferred emoji sequences that used only two units to those with three units, but no specific categorical patterns exceeded the threshold of significance. Only the rate of SVO sequences appeared to come close to this threshold, but did not exceed it. In addition, differences in frequency between patterns were minimal. Some evidence of the influence of participants’ spoken languages was found, given the higher (but not significant) rates of SVO orders, and correlations with language experience. Such results could be attributed to the prevalence of SVO in participants’ spoken languages (Vastenius et al., 2016), that the experimental languages of English and Dutch used in the experiment primed them for SVO orders cross-modally (i.e., text to emoji), or that participants used an established lexicon rather than improvised forms (Marno et al., 2015). Nevertheless, the overall infrequency of such categorical utterances leads us to treat such findings with caution, and they further suggest that, despite such possible influence, they play a more limited role in emoji communication overall.

Given the greater frequency of linear grammars, and the relative infrequency of sequences using categorical roles, it suggests further that emoji are not using complex aspects of sequencing. Categorical roles are typically orthogonally introduced at higher levels of the Jackendoff and Wittenberg (2014) hierarchy, along with simple phrase grammars. Here, simple phrase grammars were used with surprising frequency, and only with a single level of embedding. In addition, such embedding was often used to create single units in an otherwise simple sequence (Table 1) or to embed one type of linear grammar into another, such as reduplication into a semantic list (Fig. 3a). Thus, overall it appears that emoji sequencing remains in fairly low levels of the grammatical hierarchy.

Finally, it is worth noting the ‘success’ that participants had in communicating with only emoji. Although participants’ ratings for difficulty of comprehending and creating emoji-only sequences were fairly average (~ 3 on a five-point scale), the gist of only 17.5% utterances overall were annotated as being misinterpreted. This rate persisted despite the somewhat heterogenous character of our sample in terms of gender (predominately female) and diverse cultures, which could potentially skew the interpretation of emoji. This suggests that comprehension and creation of emoji-only utterances may be fairly demanding, but overall they can be fairly comprehensible.

## Experiment 2: multimodal interactions

Although emoji sometimes appear in isolation, they more often occur in multimodal interactions with text. In the second task of our experiment, we examined the ways in which emoji interact with the grammar in written language. As discussed above, here we find a useful symmetry between the relations of text and emoji to speech and gesture. Like gesture, emoji can accompany a sentence (concurrent) or be substituted into its syntax (component). Although we were also interested in the former type of interactions, we were curious specifically about how emoji might directly interface with verbal syntactic structure, as in substitutions. Our task thus specifically focused on these substitutive interactions by asking participants to purposefully insert emoji into their sentences. Emoji substitution has been observed to be less frequent than those occurring outside a sentence (Cramer et al., 2016; Duerscheid & Siever, 2017; Kelly & Watts, 2015; Markman & Oshima, 2017; Na'aman et al., 2017; Zhou et al., 2017), and thus our elicitation aimed to push this type of production specifically to observe what occurs when this type of emoji-grammar interaction occurs.

Several studies have looked at the processing of images that have been inserted into sentence contexts. Potter, Kroll, Yachzel, Carpenter, and Sherman (1986) showed participants sentences with images that were congruous or incongruous with their substituted nouns with a rapid presentation rate. Overall, substitutions were only marginally more difficult to comprehend and remember than sentences with all words. More recent work showed that substitution of nouns and verbs for images in sentences can be interpreted close to the accuracy of fully verbal sentences (Mihalcea & Leong, 2008). However, substitution was most effective for shorter and syntactically less-complex sentences, and when accompanying high-frequency words. Such results overall imply an integrated system for the processing of meaning in both words and images.

Such interpretations are further supported by additional research examining substituted images in the nouns of sentences while measuring participants’ electrophysiological brain activity (event-related brain potentials). Such work has shown that substitution of incongruous images for words elicits neural responses indicating greater semantic processing (N400) than congruous substituted images, similar to the brain response to incongruous or unexpected words (Federmeier & Kutas, 2001; Ganis, Kutas, & Sereno, 1996; Nigam, Hoffman, & Simons, 1992). Such results imply that, despite the modality switch, sentence contexts can modulate the semantic processing of images. It is worth noting that the inverse effect also occurs for words substituted for images in a visual narrative sequence (Manfredi, Cohn, & Kutas, 2017). Thus, substitutions of one modality into the grammatical structure of another occurs across domains, suggesting a fluidity between how meaning is expressed by different modalities, while negotiating the dominant structure of only one (Cohn, 2016).

Overall, messages with emoji substituting for words take readers more time to read than textual messages (Gustafsson, 2017), and can be modulated by sentence context, such as its semantic congruity for describing events (Madden & Therriault, 2009). This led us to ask in previous work whether emoji substituted into sentences interact with their grammatical structure, in addition to their semantics. We found that emoji substituted into sentences in both noun and verb positions evoked longer self-paced reading times compared with the substituted words (Cohn, Roijackers, Schaap, & Engelen, 2018). However, these congruous substitutions were read faster than emoji substitutions that were switched from their presumed grammatical types (i.e., noun emoji in verb position, or vice versa). In addition, switched emoji also incurred costs at the word following the substitution, unlike the words after congruous emoji. This suggested that emoji have preferential ways in which they interact with the grammar of sentences.

While these prior findings suggest that emoji interact with both semantic and grammatical structure, few studies have examined the nature of emoji substitutions in production. We therefore questioned which grammatical and semantic categories are most likely to be replaced in naturalistic multimodal digital communication, along with our other questions about the patterning of emoji placed into sentence structures. Given the prior literature, we predicted that nouns would be substituted the most, possibly followed by verbs.

### Methods

#### Participants

Experiment 2 included the same participants as Experiment 1.

#### Procedure

Within the four rounds of the overall study, the fourth round specifically aimed to elicit multimodal interactions here reported in Experiment 2. In this round, both participants were allowed to use written words, but were asked to substitute at least one emoji for words in their sentences. In Experiment 2, we report substitution results from round 4. In addition, because the nonemoji-only conversationalist in rounds 1–3 could have also produced emoji with their textual utterances, we also report on all emoji production from nonemoji-only participants from rounds 1–3 here in Experiment 2.

#### Annotation

Our analysis of the relations between emoji and sentence grammar focused on where substitution occurred, what was being substituted, and any additional structure it displayed. A given emoji could be assigned more than one classification if it satisfied numerous criteria. We coded the position of the emoji relative to the text, whether it was at the end of a sentence, substituted within a sentence, or substituting for the whole sentence. Note that it was also possible for these to occur at the same time, such as a sentence both with an emoji substituted for a word and placed at the end of it.

For emoji that were substituted for words, we coded their grammatical function (subject versus object), their grammatical category (noun, verb, adjective, adverb, preposition, determiner), and their semantic category (inanimate object, animate object, location, action, property, path). Confirmation of these designations were made using participants’ annotations of their own conversations. We also coded the semantic relationship of emoji to the rest of the sentence. An emoji was ‘redundant’ if the meaning of the emoji also occurred in the text, while it was ‘extratextual’ if the meaning was not mentioned in the text (Donato & Paggio, 2017). ‘Associative’ emoji connected to the broader semantic field evoked by a sentence. ‘Metonymies’ again used an emoji related to the meaning trying to be conveyed (e.g.,  for the event of graduating).

Finally, additional structural or pragmatic traits of emoji were coded, following the criteria in Experiment 1. We noted if emoji followed properties of one-unit grammars (formulaic expressions, responsive emotions) or linear grammars (temporal sequences, unrelated or semantic lists, reduplication).

#### Data analysis

Our analysis of the interactions between syntax and emoji focused on utterances produced by the nonemoji-only conversationalists in Experiment 1 (experimental rounds 1–3, for multimodal production not specifically asked for), and the utterances generated in the final, fourth round, here partitioned as Experiment 2 (for task-elicited multimodal production). In this round, participants were asked to use text, but also to substitute at least one emoji into each sentence. We again used a subjects-analysis that calculated a mean for each coding under analysis divided by the total number of utterances for each participant, and compared means across participants.

As in Experiment 1, we used one-sample *t*-tests to assess whether the rates of our categories reached a significant threshold. Here, we compared means against the chance occurrence that it could appear out of the total possibilities of that type of category. For example, in the case of grammatical categories, substitutions were compared against the 1/6 (.167) chance that it could have been assigned to one of the six coded parts of speech. These thresholds thus varied for semantic categories (1/6: .167), semantic relationships (1/4: .25), structural properties (1/6: .167), and emoji position (1/3: .33).

For our analysis of grammatical and semantic categories, we calculated each participant’s rate of substituting a particular category out of the total rate of occurrences of that category (e.g., total number of substituted emoji nouns out of the total number of nouns—emoji or words—produced by a participant). This was conducted because some categories may appear at different rates than others (e.g., a transitive sentence uses two nouns but one verb). We then compared our data using ANOVAs across groups of categories, followed by post-hoc comparisons using a Bonferroni correction.

### Results

We analyzed 152 utterances with multimodal interactions between sentences and emoji. Participants averaged 1.66 (SD = 0.51) emoji per utterance. Substantially more emoji were produced in utterances that were substituted for words (M = 0.69, SD = 0.28) than those at the end of sentences (M = 0.23, SD = 0.28) or as isolated utterances (M = 0.11, SD = 0.18). Those within sentences occurred at a rate greater than chance (.33; *t*(15) = 5.2, *p* < .001), while those as isolated utterances appeared less frequently than chance (*t*(15) = −5.1, *p* < .001). Those at the end of sentences did not differ from chance (*p* = .193).

**Fig. 4:**
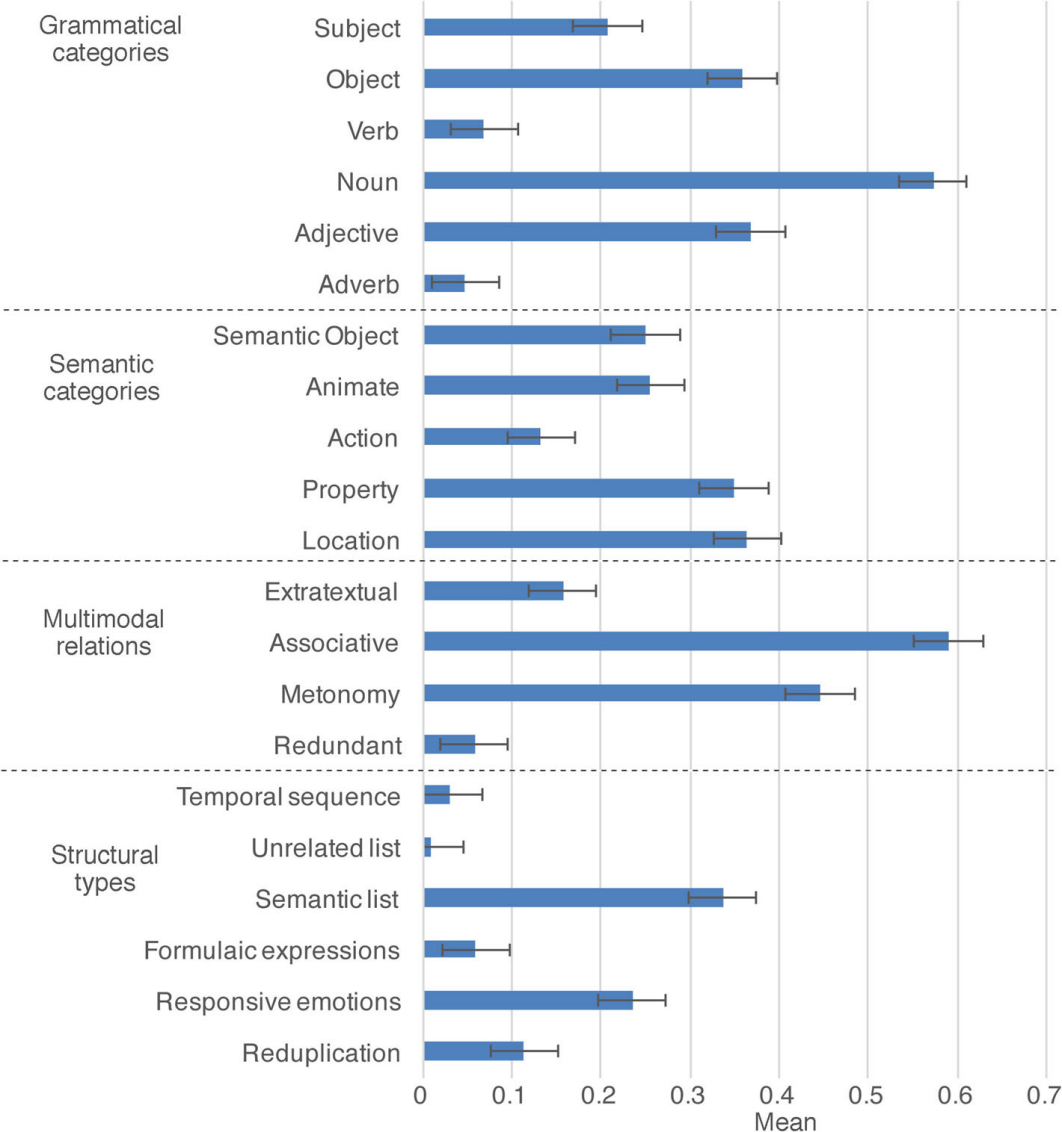
Characteristics of multimodal interactions between emoji and text, primarily those substituted into sentences for words. Error bars depict standard error

All results for characteristics of emoji in Experiment 2 are provided in Fig. 4. Emoji that were substituted for nouns and adjectives both appeared at a rate greater than chance (.167; all *t* > 2.8, all *p* < .05), while verbs and adverbs were substituted less than chance (all *t* < −4.6, all *p* < .001). No prepositions or determiners were substituted and were thus omitted from our analyses. Although nouns were used more than all other substitutions, they did not differ in the rates that they appeared as either grammatical subjects or objects (*p* = .105). Grammatical categories differed in the rates they were substituted; F(3,45) = 25.5, *p* < .001. Nouns and adjectives were used more than both verbs and adverbs (all *p* < .01), but these pairs did not differ.

No semantic categories were used higher than the threshold of chance (.167), although trends towards significance were observed for properties (*p* = .066) and locations (*p* = .082). These rates of substitution for semantic categories also did not differ from each other (*p* = .399).

In multimodal contexts, we observed significant rates of emoji that were associated with the text through a common semantic field or a metonymic connection (all *t* > 3.9, all *p* < .005). However, meanings that were directly redundant to those in the text occurred less than chance (.25; *t*(15) = − 7.7, *p* < .001), and those that were unrelated to the textual meaning trended towards significance as less frequent than chance (*p* = .069). These rates also differed from each other; F(3,45) = 31.7, *p* < .001. This occurred because associative relations appeared significantly more than metonymic relations (*p* < .001), which in turn were more frequent than extratextual relations (*p* < .01), which were more frequent than those redundant with text, although this contrast only approached significance (*p* = .074).

No structural traits (formulaic expressions, responsive emotion, lists, and so on) exceeded the threshold of chance (.167). However, these frequencies did differ from each other; F(5,75) = 12.67, *p* < .001. This occurred because semantic lists appeared more frequently than all other structural types except responsive emotions (all *p* < .05), which appeared more frequently than temporal sequences and unrelated lists (*p* < .05) but not formulaic expressions or reduplication. Reduplications, in turn, were more frequent than unrelated lists (*p* < .05).

### Discussion

This experiment examined the multimodal interactions of emoji with sentence structures. In this task, participants were asked to substitute at least one emoji per sentence into their utterances. The high proportion of utterances (69%) with substituted emoji confirm that they followed this instruction, though they also, uninstructed, added emoji at the end of their sentences (23%) and sometimes responded in emoji-only utterances (11%). Such proliferation suggests that, at least in the context of our experiment, participants were comfortable using emoji to communicate beyond just our requested experimental task. This is also evident in that these means add up to above 100%, meaning that emoji were appearing in more than one position per utterance.

When emoji were substituted into sentences, participants used them only to replace content words and not function words, consistent with prior corpus work (Na'aman et al., 2017). Within these substitutions, emoji appeared more as nouns than any other grammatical category, and more as the grammatical objects than subjects of sentences. Surprisingly, emoji were also often used as adjectives. The most frequent emoji adjective used the thumbs-up emoji, as in “the  zoo”, annotated as “the best zoo”. Far fewer emoji were substituted for verbs or adverbs, which is consistent with Potter et al.’s (1986) speculation that verbs would be less natural to substitute with images than nouns, because pictures of actions also depict the objects doing those actions. Nevertheless, prior work showed no difference between the reading times of noun-emoji versus verb-emoji substitutions into sentences (Cohn et al., 2018). Such findings suggest that verbs may not be optimal for substitutions, though they are comprehensible so long as they remain congruous within a sentence.

One reason posited for a hypothetical prevalence of images substituting for nouns over verbs was the idea that concrete objects are easier to show in images (Potter et al., 1986). However, our coding of semantic categories did not show a larger number of substitutions for concrete concepts (semantic objects, animate objects). Although only approaching significance in their rate of occurrence, we did observe several substitutions expressing nonconcrete properties. For example, one participant used  to express someone being “creative”, or  as a positive property.

Finally, these emoji connected multimodally with text in ways that expanded or elaborated on the meaning more than being just redundant. Multimodal relations primarily used connections to a broader semantic field or metonymy, not connections redundant with the text or fully disconnected from it. This is consistent with corpus data from Twitter suggesting emoji are used to add new information more often than repeating information in sentences (Donato & Paggio, 2017). This suggests that substitutive emoji are being used in a way that is consistent with the semantics of their replaced words or, barring that directly, metonymically associated.

## General discussion

We here examined how emoji take on and/or interface with grammatical structures when generated in interactive conversations. Following studies on gesture-only research (Gershoff-Stowe & Goldin-Meadow, 2002), we asked participants to communicate using only emoji to maximize the potential for systematic patterns, and also to substitute emoji into their sentences to see their interaction with syntax.

Overall, we found evidence of only simple sequencing patterns, typically at the level of ‘linear grammars’, with comparatively reduced use of categorical roles and embedding (Jackendoff & Wittenberg, 2014). In addition, participants used minimal consistency of sequencing of categorical roles across utterances. Finally, we found that emoji can readily be used to substitute for words in sentences but are chosen to substitute more for nouns or adjectives than verbs, and more for grammatical objects than subjects. It is also worth noting that, although our experiment elicited far fewer utterances than the thousands or millions analyzed in large-scale digital corpora (Duerscheid & Siever, 2017; McCulloch & Gawne, 2018; Na'aman et al., 2017), we observed trends consistent with those works. Such consistency offers further support for the robustness of these basic level sequencing patterns. In addition, we believe our experiments add analytical nuance to the study of emoji sequencing, which would be informative in the analysis of larger corpora.

Altogether, these results imply that the sequential patterning that people use when communicating with emoji has minimal grammatical complexity. Rather, our analysis implies that they use sequencing patterns more consistent with simple, linear grammars that rely on their properties of semantic relations. However, this begs the question, given the rapid proliferation and prevalent usage of emoji in daily communication, could they develop more complex grammatical structures in the future? Indeed, cases of languages ‘emerging’ for a community have occurred in the bodily modality, most famously for Nicaraguan Sign Language. Here, deaf individuals with unique gesture systems created a shared lexicon through social interactions, which added greater complexity as successive generations of speakers refined the system (Kegl, 1994; Senghas, 1986). Could this also happen to create a more complex ‘emoji language’? We find this highly unlikely for two reasons.

First, the possibility for combinatoriality in emoji is limited by their nature as codified whole-unit signs. The emoji lexicon is provided to users within their software applications, and this lexicon is governed by sources external to the users themselves (i.e., the Unicode Consortium). Users have no access to basic primitives that constitute emoji (such as the graphemic shapes of letters and punctuation that at least comprise emoticons), and can only use the whole units that the lexicon provides. As a result, users cannot create new emoji, nor can they alter, manipulate, or combine the emoji that currently exist in any easy, efficient, or productive way. Modulation of an emoji requires encoding a fully different lexical entry into the emoji system, such as variants of skin color for emoji faces. For further complexity to emerge, users would need to be afforded greater creativity to manipulate or combine emoji in productive ways.

In contrast, actual drawing systems allow for fairly free-form combinations between schematic forms (Cohn, 2012; Wilson, 1988)—such as altering eyes or mouths on a face—while emoji allow for limited internal combinations, such as affixation discussed above. A good example of this is the 2016 Word of the Year chosen by the American Dialect Society: “Dumpster fire”. This has a rebus emoji analog that itself is noncompositional with . Emoji cannot place the fire onto the dumpster while staying within the same line of text, with the fire spatially on top of the trashcan, as would occur in an actual drawing. Thus, emoji force relational concepts to be presented in a linear sequence, rather than allowing them to use the affordances of the visual modality to spatially combine visual parts together in a natural way. Emoji are hamstrung by their constraint to act like text, which forces a fairly unnatural linear sequence for graphic communication. Without the basic ability for units themselves to be combinatorial, we do not believe that further complexity is likely to emerge.

Second, we believe that image sequences are not optimal for the ‘sentence level’ of meaning, because such semantic information could be captured in a single image. Roles like nouns and verbs may thus not be natural for the visual modality, in contrast to the fairly natural categorical roles used for sequencing visual images at higher levels of information structure, namely narrative categories (Cohn, 2013b, 2014). Indeed, visual communication typically organizes information at a larger, narrative level, which does seem to involve architectural principles similar to linguistic syntax (Cohn, 2013b), and its manipulation evokes similar brain responses, as measured using event-related potentials, as violations of syntax (Cohn et al., 2012; Cohn et al., 2014).

Thus, if visual images are optimized for a greater information level, emoji—and any low-level semantic signs—will thus always be limited in their sequencing, because they force visuals to operate in a way that is unnatural to their graphic modality. Indeed, pictorial systems with robust conventionality and combinatoriality already appear in the graphic system of the world, and are argued to constitute diverse ‘visual languages’ (Cohn, 2013a). In contrast, emoji are structurally limited, despite their useful role in interactive communication. This is somewhat analogous to efforts to use Manually Coded English which unnaturally imitates the properties of spoken English in the manual modality, which skirts the natural properties of an actual sign language (e.g., Supalla, 1991).

Thus, emoji are limited in their combinatoriality, rely on a lexicon provided and mediated by external sources, and constrain information to an unnatural level of information structure for visual communication. For these reasons, we believe that emoji are structurally constrained by their technology from further developing more robust linguistic structures to become full ‘languages’, despite the rich interactive social environment in which they are used. This does not mitigate the clear communicative benefits of emoji in interactive multimodal exchanges, but it is a reminder that modalities of expression operate with different affordances connected to the properties of the modalities themselves. While processing mechanisms appear to be shared across the verbal and visual domains (Cohn et al., 2012; Cohn et al., 2014; Weissman & Tanner, 2018), it does not mean that they manifest in modality-independent ways.

Overall, this work has extended the hierarchy of grammars from the sequencing of spoken and bodily communication systems (Jackendoff & Wittenberg, 2014, 2017) to that of graphic communication, possibly suggesting a range of domain-general sequencing strategies available to human cognition (Dehaene et al., 2015). In the case of emoji-only sequencing, they appear to lack the characteristics of complex grammar, instead relying on linear patterning motivated by the meanings of the emoji themselves. However, neither the graphic nor verbal modality naturalistically appears in isolation, even when imposing experimental constraints to do so. In line with this, emoji appear to be effective for communicative multimodal interactions, often using text and image relationships similar to those between speech and gestures. These expressions are useful and enhance communication. Such findings further reinforce that restricting the perspective of research to a single modality—such as just verbal language or just graphics—limits the understanding of the structural properties of those modalities themselves and of their interactions in the full scope of human communication.

## Data Availability

The datasets used and/or analyzed during the current study are available from the corresponding author on reasonable request.

## Footnotes

1 Jackendoff and Wittenberg (2014) first called these concatenation grammars, but revised the terminology in subsequent publications (Jackendoff & Wittenberg, 2017).
